# Nicotine-Like Effects of the Neonicotinoid Insecticides Acetamiprid and Imidacloprid on Cerebellar Neurons from Neonatal Rats

**DOI:** 10.1371/journal.pone.0032432

**Published:** 2012-02-29

**Authors:** Junko Kimura-Kuroda, Yukari Komuta, Yoichiro Kuroda, Masaharu Hayashi, Hitoshi Kawano

**Affiliations:** Department of Brain Development and Neural Regeneration, Tokyo Metropolitan Institute of Medical Science, Setagaya-city, Tokyo, Japan; Sanford-Burnham Medical Research Institute, United States of America

## Abstract

**Background:**

Acetamiprid (ACE) and imidacloprid (IMI) belong to a new, widely used class of pesticide, the neonicotinoids. With similar chemical structures to nicotine, neonicotinoids also share agonist activity at nicotinic acetylcholine receptors (nAChRs). Although their toxicities against insects are well established, their precise effects on mammalian nAChRs remain to be elucidated. Because of the importance of nAChRs for mammalian brain function, especially brain development, detailed investigation of the neonicotinoids is needed to protect the health of human children. We aimed to determine the effects of neonicotinoids on the nAChRs of developing mammalian neurons and compare their effects with nicotine, a neurotoxin of brain development.

**Methodology/Principal Findings:**

Primary cultures of cerebellar neurons from neonatal rats allow for examinations of the developmental neurotoxicity of chemicals because the various stages of neurodevelopment—including proliferation, migration, differentiation, and morphological and functional maturation—can be observed *in vitro*. Using these cultures, an excitatory Ca^2+^-influx assay was employed as an indicator of neural physiological activity. Significant excitatory Ca^2+^ influxes were evoked by ACE, IMI, and nicotine at concentrations greater than 1 µM in small neurons in cerebellar cultures that expressed the mRNA of the α3, α4, and α7 nAChR subunits. The firing patterns, proportion of excited neurons, and peak excitatory Ca^2+^ influxes induced by ACE and IMI showed differences from those induced by nicotine. However, ACE and IMI had greater effects on mammalian neurons than those previously reported in binding assay studies. Furthermore, the effects of the neonicotinoids were significantly inhibited by the nAChR antagonists mecamylamine, α-bungarotoxin, and dihydro-β-erythroidine.

**Conclusions/Significance:**

This study is the first to show that ACE, IMI, and nicotine exert similar excitatory effects on mammalian nAChRs at concentrations greater than 1 µM. Therefore, the neonicotinoids may adversely affect human health, especially the developing brain.

## Introduction

The neonicotinoids acetamiprid (ACE) and imidacloprid (IMI) belong to a new class of insecticides that are used worldwide to protect crops from pest insects and domestic animals from fleas [1]. The neonicotinoids have been reported to act as agonists of nicotinic acetylcholine receptors (nAChRs), and their high toxicities to insects have been attributed to selective binding affinity to insect nAChRs [2], [3]. Furthermore, X-ray crystallography has revealed that the binding sites of the neonicotinoids on nAChRs are electronegative, which contributes to their characteristic toxicities at insect nAChRs [4]–[6]. However, X-ray crystal analyses and binding assays against one type of nAChR have often yielded controversial results, and the structural conformations of receptors are often changed by physiological actions such as ligand binding or interactions with other proteins [7].

There have been a few studies of neonicotinoid-induced toxicity in the nervous systems of vertebrates, and these studies were conducted with only a few of the neonicotinoids, such as IMI, thiamethoxam, and clothianidin. IMI has been reported to act as an agonist or an antagonist of nAChRs at 10 µM in rat pheochromocytoma (PC12) cells [8] and to change the membrane properties of neurons at ≥10 µM in the mouse cochlear nucleus [9]. Exposure to IMI *in utero* causes decreased sensorimotor performance and increased expression of glial fibrillary acidic protein (GFAP) in the motor cortex and hippocampus of neonatal rats [10]. Furthermore, it has been reported that the neonicotinoids thiamethoxam and clothianidin induce dopamine release in the rat striatum via nAChRs [11] and that thiamethoxam alters behavioral and biochemical processes related to the rat cholinergic systems [12]. Recently, IMI and clothianidin have been reported to agonize human α4β2 nAChR subtypes [13]. These findings suggest that the neonicotinoids affect mammalian nAChRs to a greater extent than previously believed based on binding-assay data and that further study of the neonicotinoids is needed to protect human health. The relevance of neonicotinoid exposure to human health is especially important in children because nAChRs are important for normal brain development [14] and their functions are disturbed by nicotine [15]. Therefore, the effects of neonicotinoids on mammalian developing neurons should be investigated and compared with those of nicotine, as a positive control that is known to affect mammalian nAChRs.

In the present study, we chose the globally used neonicotinoids ACE and IMI. Importantly, ACE has relatively higher affinities for rodent nAChRs than other neonicotinoids [16], and little published information is available regarding its effects on mammalian nAChRs. We compared the effects of ACE and IMI on mammalian nAChRs with those of nicotine, using an excitatory Ca^2+^-influx assay as an index of neural physiological activity. We conducted these assays in cultured cerebellar neurons from neonatal rats, which natively express the α3, α4, and α7 nAChR subtypes *in vitro* [17]. Primary neonatal cerebellar neurons have been used as a suitable model to evaluate developmental neurotoxicity because they allow for the observation of the various stages of neurodevelopment, including proliferation, migration, differentiation, and morphological and functional maturation [18]. The overall aim of this study was to determine whether these two neonicotinoids exert effects similar to those of nicotine on cerebellar neurons from neonatal rats.

## Results

### Cerebellar cultures and expression of nAChR mRNA

**Figure 1:**
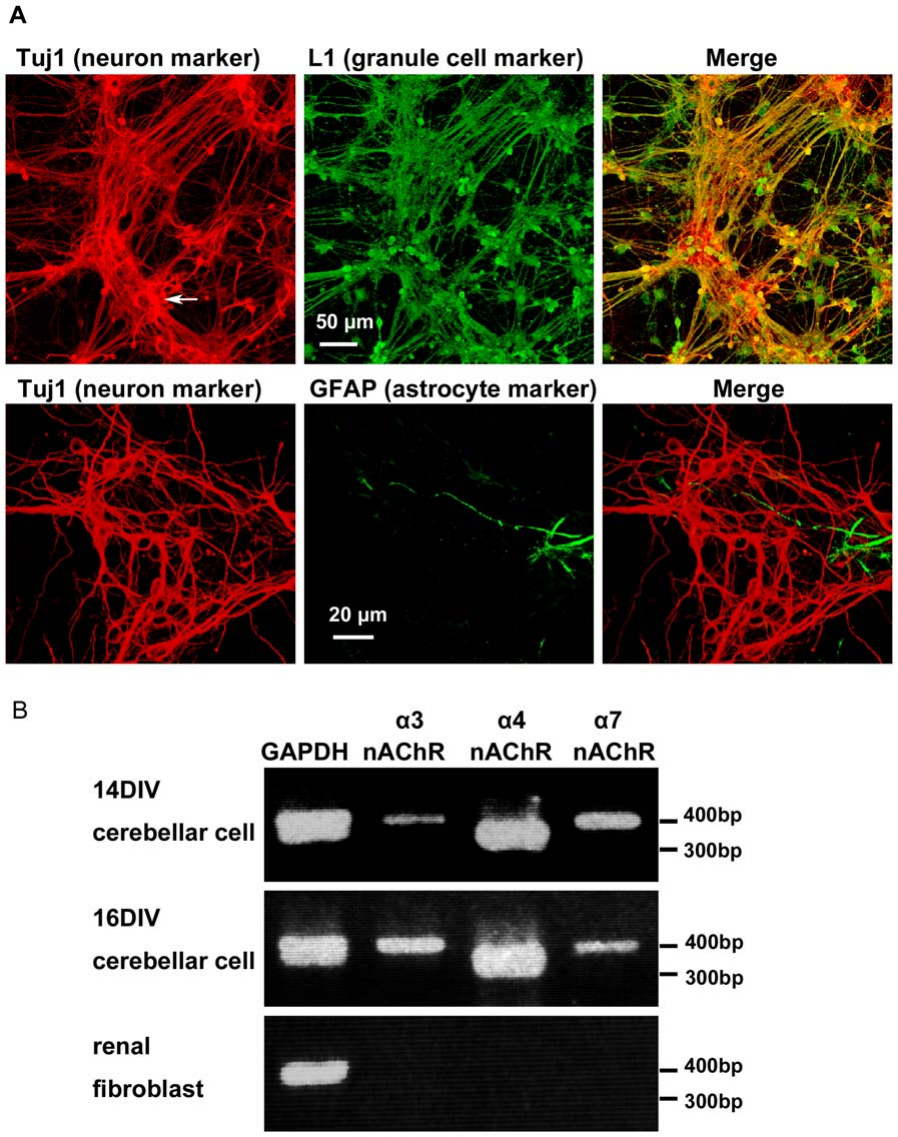
Cerebellar cell culture at 14 DIV and RT-PCR of nAChR subunits. (**A**) As can be seen in the upper panels, most of Tuj1-positive small neurons were immunoreactive for L1, which indicates that they are granule cells. The large Tuj1-positive neurons (arrow) are Purkinje cells, based on their shape and size. As can be seen in lower panels, the Tuj1-positive neurons and GFAP-positive astrocytes are clearly distinct. (**B**) RT-PCR of nAChR subunit transcripts in cerebellar cultured cells and renal fibroblast cultures. The mRNA transcripts for the α3, α4, and α7 nAChR subunits were amplified from cerebellar cultures at 14 and 16 DIV and from renal fibroblasts. GAPDH was used as a positive control. Products of the predicted size were sequenced to confirm their identity. The α3, α4, and α7 nAChR subunits were expressed in cerebellar cells but not in kidney fibroblasts.

In cerebellar cultures from neonatal rats at postnatal day 1 (P1), approximately 90% of the total cells were small neurons stained by anti-Tuj1 (neuron specific β3-tubulin). Almost all of these small neurons were identified to be cerebellar granule cells based on their morphology and that they were stained by anti-neural cell adhesion molecule L1 (Fig. 1A) [19]. The cerebellar cultures also contained a few large, Tuj1-positive Purkinje neurons (about 1%, arrow in Fig. 1A) and GFAP-positive astrocytes (about 5%, Fig. 1A). Because most commercially available antibodies against each of the subunits of nAChRs have been reported to be cross reactive to other subunits or unknown factors [20], we examined the mRNA of nAChR subunits with RT-PCR. As shown in Fig. 1B, mRNAs of the α3, α4, and α7 nAChR subunits are expressed in cerebellar cells at 14 and 16 days *in vitro* (DIV), which was the time frame used for the Ca^2+^-influx assay. The α4 nAChR subunit was expressed constantly at 14 and 16 DIV. The expressions of the α3 and α7 nAChR subunits showed little difference between 14 and 16 DIV. In renal fibroblast cultures, however, mRNAs of the α3, α4, and α7 nAChR subunits were not expressed.

### Ca^2+^ influx in cerebellar neurons

**Figure 2:**
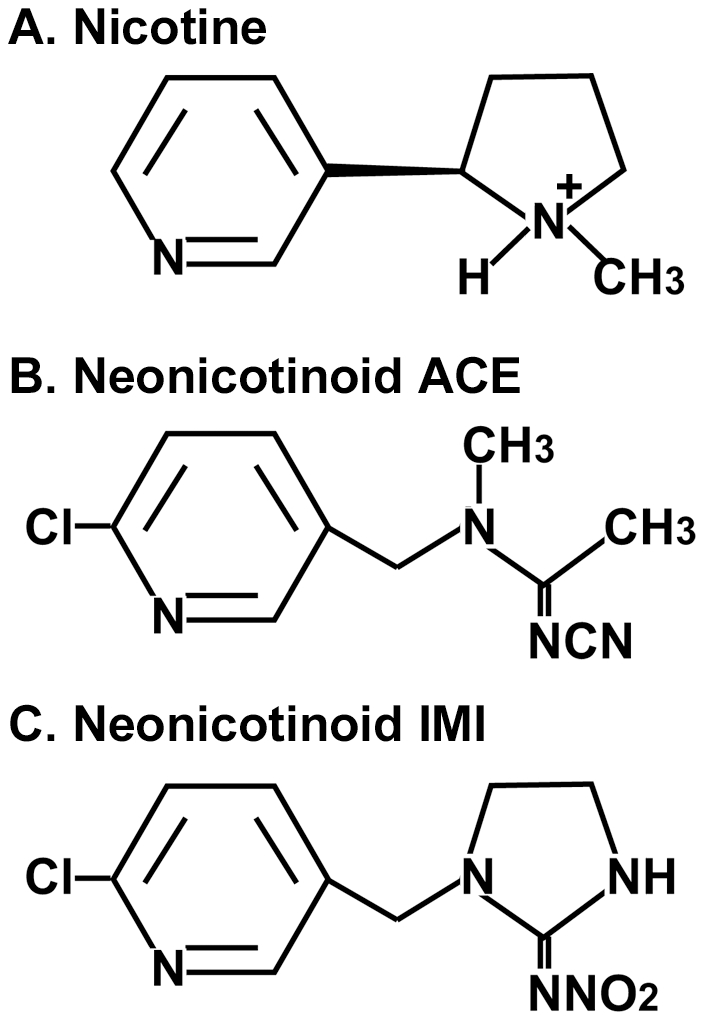
Molecular structures of nicotine and the neonicotinoids ACE and IMI. (**A**) nicotine, (**B**) acetamiprid (ACE), (**C**) imidacloprid (IMI).

**Figure 3:**
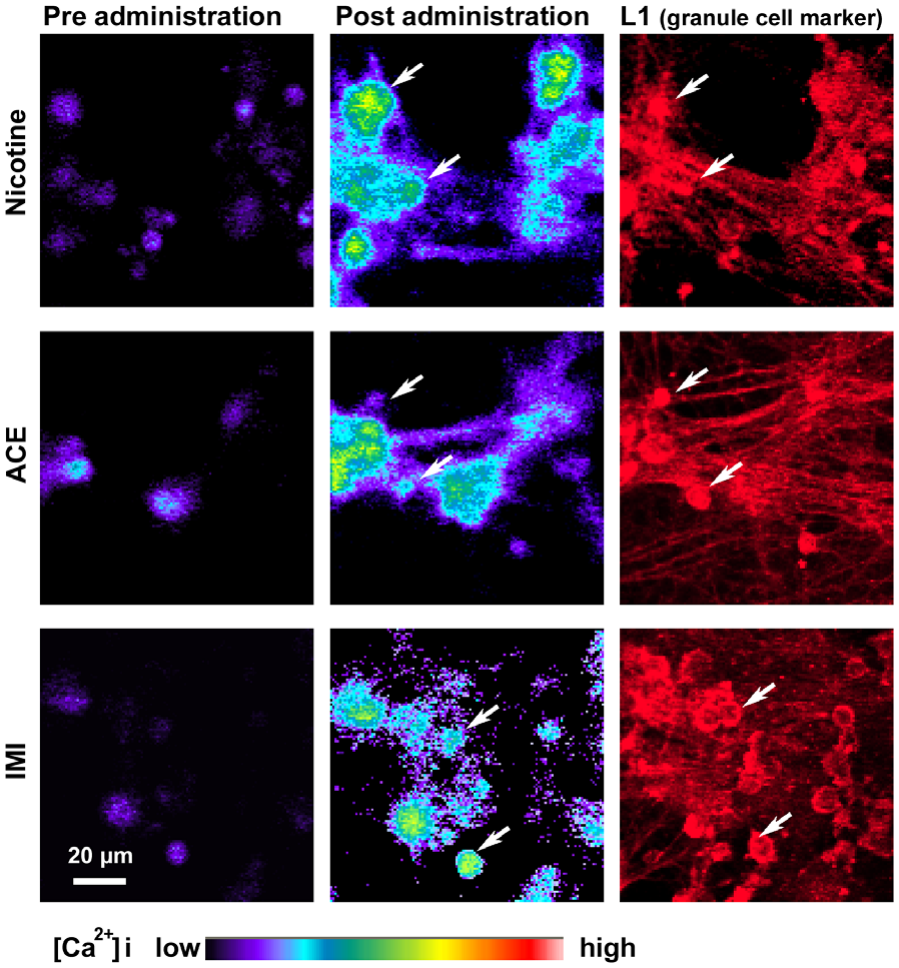
Ca^2+^ influx in cerebellar small neurons. Excitatory Ca^2+^ influxes in small round-shaped neurons (14 DIV) were observed just after the administration of nicotine, ACE, or IMI at 10 µM. The left column shows the time period before application of the drug, and the middle column shows the time period immediately after application of the drug. The pseudo-color bar in the bottom indicates the Fluo-4 fluorescence intensity scale. The right column shows L1-immunoreactivity from the same field of the Fluo-4 experiment. The arrows indicate round small neurons that were immunoreactive for L1.

To determine the effects of neonicotinoids on the nAChRs of cerebellar neurons, we examined intracellular Ca^2+^ mobilization using the calcium-sensitive fluorescent dye Fluo-4. The chemical structures of nicotine, ACE, and IMI are shown in Figure 2. As shown in the left column of Figure 3, Fluo-4 loading induced some fluorescence in cerebellar small neurons at 14 DIV. Applications of nicotine, ACE, and IMI at 10 µM robustly increased Fluo-4 fluorescence in round-shaped neurons (middle column of Fig. 3). We confirmed that the excited small round cells were immunoreactive for L1 (right column of Fig. 3), which is known to be a cerebellar granule cell marker [19].

A few large-sized Purkinje neurons did not exhibit significant Ca^2+^ influx following applications of ACE, IMI, and nicotine. Moreover, GFAP-positive astrocytes did not flux as much Ca^2+^ as the small neurons.

**Figure 4:**
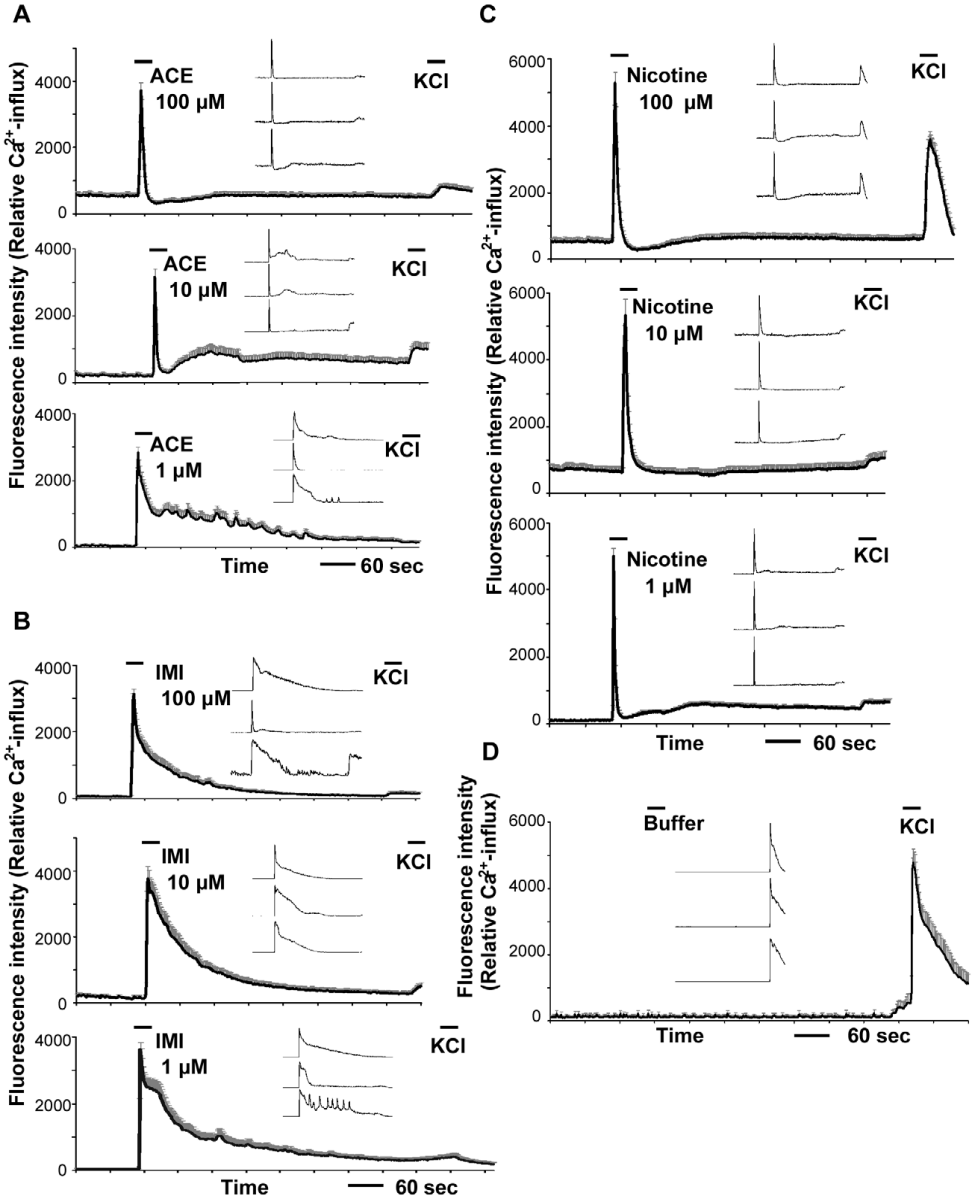
Effects of nicotine, ACE, and IMI on Ca^2+^ influxes in Fluo-4-loaded cerebellar neurons. Administration of 1, 10, or 100 µM of ACE (**A**), IMI (**B**), or nicotine (**C**) evoked a significant Ca^2+^ influx in neurons. As a negative control (**D**), BSS containing DMSO (0.001%) was applied instead of the agonists. After washing with BSS, KCl (100 mM) was added to stimulate the neurons. The main, line graphs represent mean values ± the S.E.M. of the Ca^2+^-influx-fluorescence intensities in the small neurons (*n* = 20–30). All of the main graphs show data from a series of typical experiments in at least three independent culture assays, and the same patterns of Ca^2+^ firing were confirmed. The small, line graphs inset within each main graph show the firing pattern of a single representative cell.

In Figure 4A–C, administrations of nicotine, ACE, and IMI induced a characteristic excitatory pattern of intracellular Ca^2+^ influx at 1–100 µM in small neurons. The main, line graphs represent the mean values ± the standard error of the mean (S.E.M.) (*n* = 20–30) for the fluorescence intensity of Ca^2+^ influx. The small, line graphs inset within each main graph show a representative firing pattern from a single cell. There was a rapid rise and fall in the firing patterns of these cells following applications of nicotine at 1–100 µM (Fig. 4C) and ACE at 10–100 µM (Fig. 4A). There was a rapid rise but gradual fall in the firing patterns of these cells following applications of ACE at 1 µM (Fig. 4A) and IMI at 1–100 µM (Fig. 4B). As shown in Figure 4A–C, administrations of nicotine, ACE, and IMI attenuated the responses to KCl (100 mM) washes 5–8 min after their applications compared with washes with only KCl (Fig. 4D). As shown in Figure 4C, KCl induced robust excitations after administration of nicotine at 100 µM. When 500 nM or lower concentrations of nicotine, ACE, and IMI were applied to the cerebellar cells, we did not observe significant Ca^2+^ influx during at least 3 independent replications with each drug.

### Peak value of Ca^2+^ influx and proportion of excited neurons

**Figure 5:**
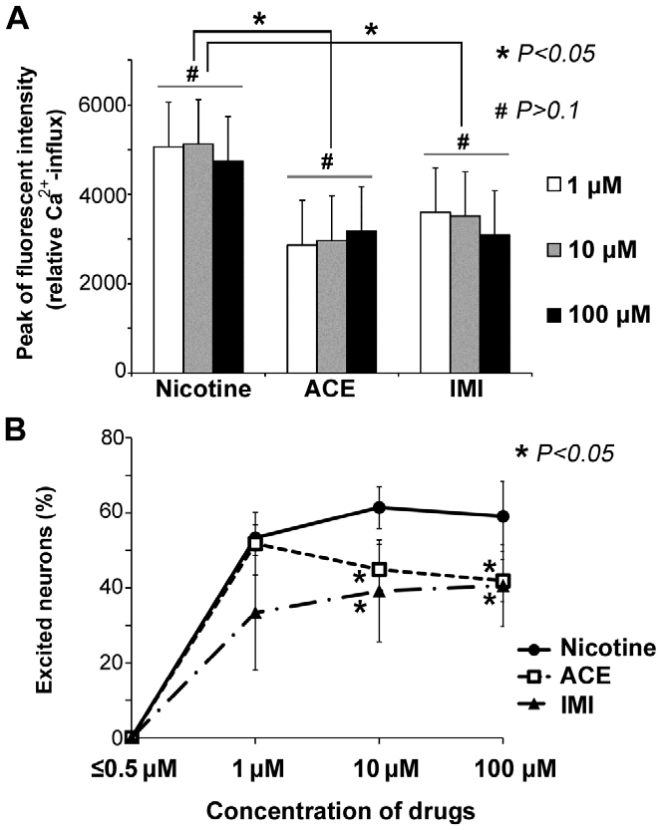
Peak of Ca^2+^ influxes and proportions of excited neurons. (**A**) Nicotine evoked higher peak Ca^2+^ influxes than ACE or IMI, whereas these two neonicotinoids showed similar peak values. The peak relative Ca^2+^ influx was calculated as the mean ± the S.E.M. of the highest fluo-4 intensities of each excited cell (*n* = 20–30), which were determined from the mean background intensity over the 60 seconds immediately before the drug application. Data within the same drug were analyzed statistically using a Student's paired t-test and no significant differences were observed (gray bar, # *P>0.1*). Data across the different drugs were analyzed by an ANOVA with a post hoc Bonferroni/Dunn test. Significant differences (* *P<0.05*) were observed between nicotine and the two neonicotinoids. All of the main graphs show data from a series of typical experiments in at least three independent culture assays, and similar values were confirmed. (**B**) The proportion of the excited neurons was compared following administrations of ACE, IMI, and nicotine at concentrations of 1–100 µM. Data within the same drug were analyzed statistically using a Student's t-test, and no significant differences were observed. Data across the different drugs were analyzed by ANOVA with a post hoc Bonferroni/Dunn test. The data represent the mean ± the S.E.M. of three to four independent experiments.

Next, we examined the peak magnitudes of Ca^2+^ influx and the dependence of the effects of the neonicotinoids on the administered dose. The peak values were compared between each concentration of the three drugs (Fig. 5A). Even at a concentration of 1 µM, ACE and IMI caused distinctive excitations in numerous small neurons, and the peak relative fluorescence intensities of Ca^2+^ influx were similar to those following applications of 10 or 100 µM of the same drug. Administration of nicotine evoked higher peaks of Ca^2+^ influx than those of ACE and IMI, and these two neonicotinoids showed similar peak values, as shown in Figure 5A.

Subsequently, we examined the proportion of cells among the total number of small neurons (1–1.25×10^3^ cells) that were excited by ACE, IMI, and nicotine. As shown in Figure 5B, the proportions of the neurons excited by nicotine were higher than those by excited by IMI. At 1 µM, ACE excited a similar proportion of the neurons to nicotine, and ACE at 10 or 100 µM excited similar proportions of the neurons to IMI.

### Antagonist assay

**Figure 6:**
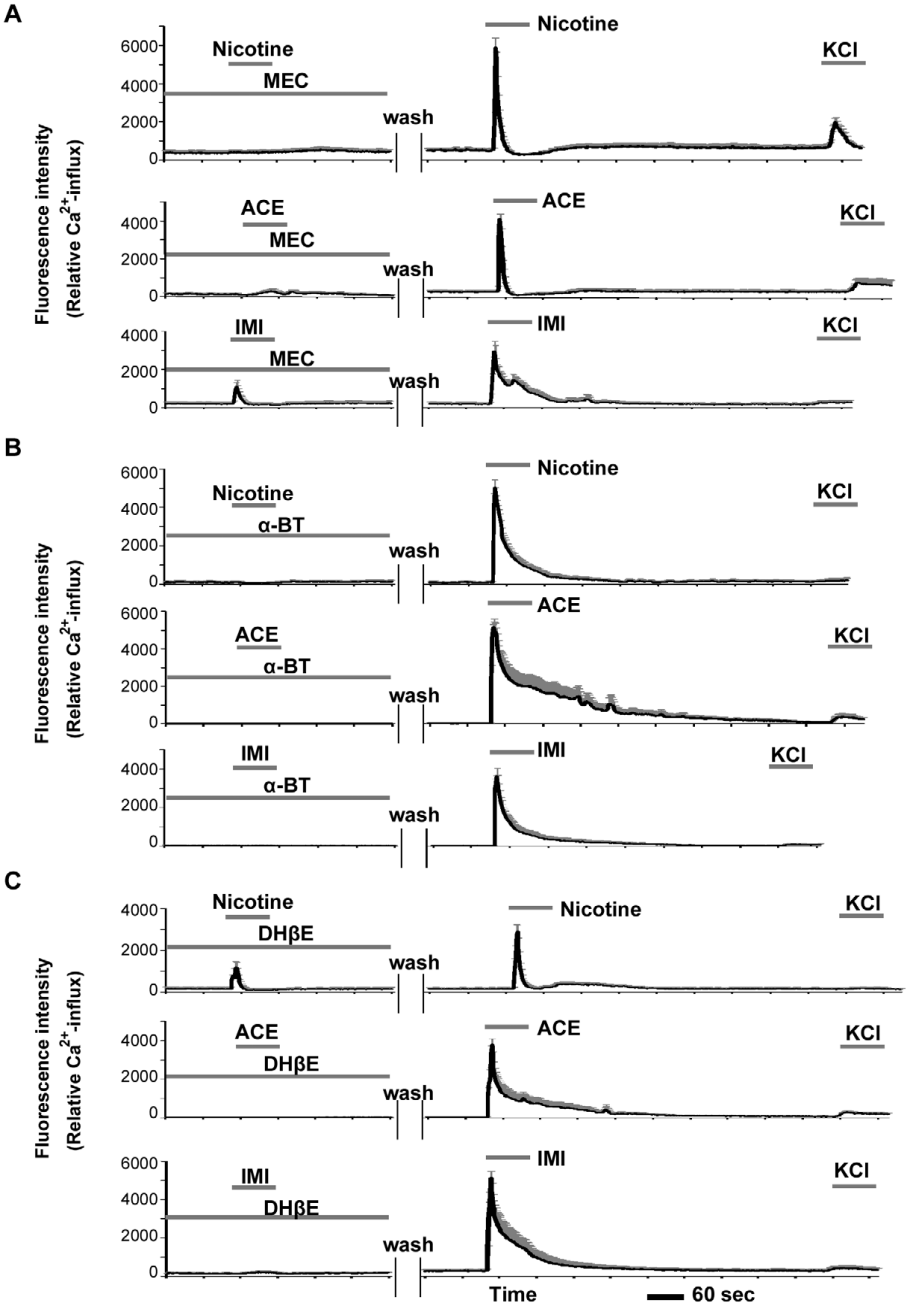
Effects of nAChR antagonists on Ca^2+^ influxes induced by ACE, IMI, or nicotine. The antagonists MEC (**A**, 100 µM), α-BT (**B**, 1 µM), and DHβE (**C**, 1 µM) all significantly inhibited the excitatory effects of nicotine, ACE, or IMI at 100 µM. MEC partially inhibited the IMI-evoked responses (**A**), and DHβE partially blocked the nicotine-evoked responses (**C**). The line graphs represent the mean ± the S.E.M. of the Ca^2+^-influx-induced fluorescence intensities in small neurons (*n* = 20–30). All of the graphs show the data from a series of typical experiments in at least three independent culture assays, and the same patterns of Ca^2+^ firing were confirmed.

The effects of neonicotinoids were also examined following pretreatments with the specific nAChR antagonists mecamylamine (MEC, nonselective nAChR antagonist), α-bungarotoxin (α-BT, selective α7 nAChR antagonist), and dihydro-β-erythroidine (DHβE, selective α4β2 and α3β4 nAChR antagonist). Pre-incubation with MEC (100 µM), α-BT (1 µM), or DHβE (1 µM) significantly inhibited the characteristic excitations and Ca^2+^ influxes in small neurons induced by nicotine, ACE, or IMI at 100 µM. Moreover, after removal of the antagonists by washes with balanced salt solution (BSS), the same neurons were excited by these agonists (Fig. 6A–C). The inhibitory effects of these antagonists showed some differences among nicotine and the neonicotinoids. As shown in Figure 6A, MEC strongly inhibited nicotine- and ACE-evoked Ca^2+^ influx but only partially inhibited IMI-evoked Ca^2+^ influx. The excitatory effects of all three agonists were completely inhibited by α-BT (Fig. 6B). Furthermore, after removal of α-BT, the nicotine- and ACE-evoked firing patterns (Fig. 5B) were rather broad compared with baseline patterns (Fig. 4A, C). DHβE partially inhibited nicotine-evoked firing (Fig. 6C) but completely inhibited ACE- and IMI-evoked firing.

**Figure 7:**
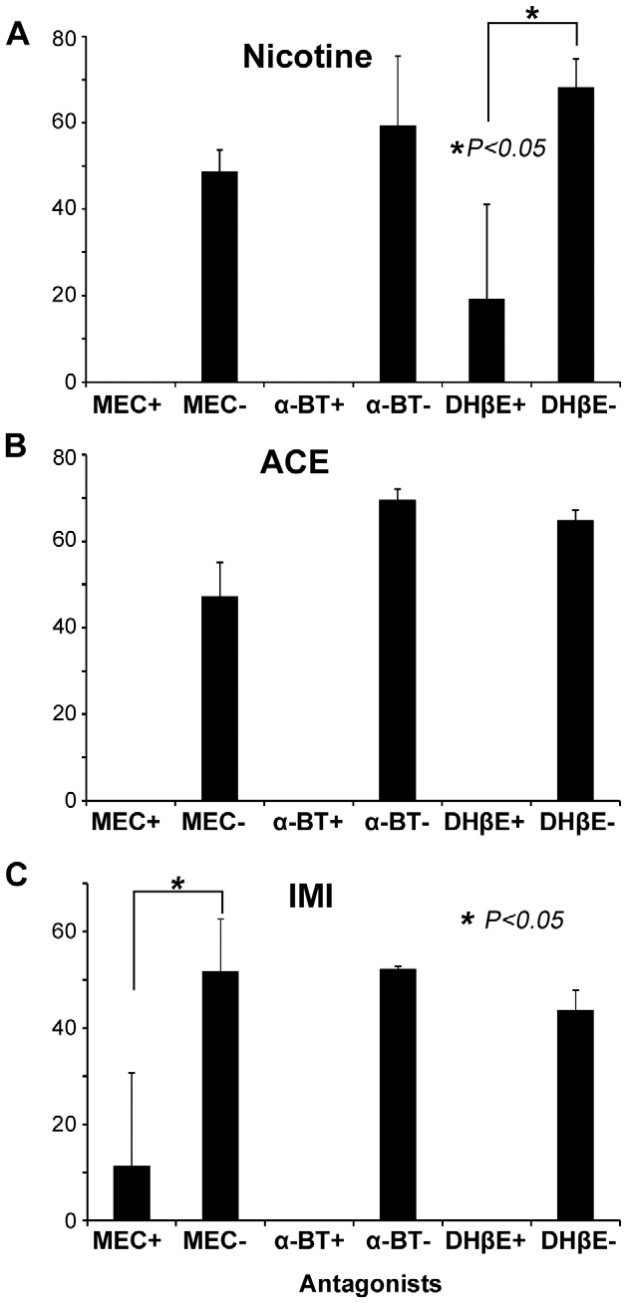
Effects of nAChR antagonists on the proportions of excited neurons. The proportions of the neurons excited by nicotine (**A**), ACE (**B**), or IMI (**C**) at 100 µM were compared in the presence or absence of MEC (100 µM), α-BT (1 µM), or DHβE (1 µM). All three antagonists significantly reduced the proportion of the cells that were excited by the agonists. The data represent the mean ± the S.E.M. of three independent experiments and were analyzed using an ANOVA followed by a post hoc Bonferroni/Dunn test.

Subsequently, we examined the proportions of the neurons that were excited by 100 µM of nicotine, ACE, or IMI in the presence or absence of each nAChR antagonist. As shown in Figure 7A–C, all three antagonists significantly decreased the proportions of the neurons that were excited by the agonists. The inhibitory potential of each nAChR antagonist showed some differences among nicotine, ACE, and IMI. Specifically, MEC and DHβE only partially inhibited the effects of IMI and nicotine, respectively.

## Discussion

### Characteristics of cerebellar neurons excited by nicotine, ACE, or IMI

In the present study, we showed that administration of either ACE or IMI at 1–100 µM evoked intracellular excitatory Ca^2+^ influxes in cerebellar neurons, which are mainly composed of granule cells (>90%). We identified these excited neurons to be granule cells, as they are small in size, round-shaped, and immunoreactive for L1 [19]. During the perinatal stage in rodents and humans, transient expression of nAChRs has been observed in the cerebellum [14]. By *in situ* hybridization histochemistry, mRNAs of α4 and α7 nAChR subunits were localized in the internal granule cell layer of P8-rat-cerebellum [21], whereas α3, α4, and α7 nAChR mRNAs were detected by RT-PCR in postnatal rat cerebellum [22] and in cultured cerebellar granule cells [17]. On the other hand, the binding sites of the nAChR agonist [^125^I]epibatidine (α3, α4 specific) and the antagonist [^125^I]α-BT were in the internal granule cell layer of P8-rat-cerebellum [21], and the binding sites of the nAChR agonists [^3^H]nicotine and [^3^H]cytisine, and the antagonist [^125^I]α-BT were detected in cultured cerebellar granule cells [17]. Based on these reports, it is highly likely that α3, α4, and α7 nAChRs were expressed in cerebellar granule cells in our cultures, and they are expressed in these cells in the developing brain.

A few large-sized Purkinje cells, which were included in the culture, did not exhibit Ca^2+^ influx following the applications of ACE, IMI, and nicotine. Previous reports have indicated that developing Purkinje cells also express α7 nAChR mRNA at P8 stage in rat [21], and α4 nAChR mRNA at fetus stage in human [23]. Excitatory or inhibitory postsynaptic currents in Purkinje cells were observed by administration of nicotine in slice culture from rats at P5–P10, however, they were not activated by nicotine in cultures from older rats [24]. The authors of those studies suggested that this lack of response was because of synaptic maturation, and this may be also the case in our cultures at 14–16 DIV from P1 rats.

Furthermore, GFAP-positive astrocytes also did not show Ca^2+^ influx following applications of ACE, IMI, and nicotine. Sustained exposure (5 min) to nicotine has been reported to induce light uptake of intracellular Ca^2+^ in cortical astrocytes [25]. In our study, we examined transient exposure to nicotine or neonicotinoids under continuous perfusion, which is likely the reason there was no response in the astrocytes.

### Similarities between the effects of neonicotinoids and nicotine on neuronal excitation

Our results indicate that at a concentration of 1 µM, ACE and IMI robustly excited rat cerebellar neurons to a similar degree as nicotine. In a previous report, using [^3^H]IMI or [^3^H]nicotine, the binding affinities of ACE, IMI, and nicotine at insect nAChRs have been reported to be 84, 565 and 0.002 times, respectively, as large as their affinities at rodent α4β2 nAChRs [2]. The discrepancy between these reported values and our results may be attributable to differences between using simplified artificial binding assays with one type of nAChR and examining cellular excitatory actions mediated by several kinds of nAChRs on a single neuron.

Previous studies have indicated that IMI and clothianidin modify the amplitude of responses to acetylcholine (ACh) by chicken or human nAChR α4β2 subtype receptors even at a low concentration (3 µM) that did not activate these receptors when administered alone [13], [26] . It is possible that the binding of ACh to nAChRs modifies the structure of the nAChRs, which may allow neonicotinoids to affect mammalian nAChRs. The present results indicate that ACE and IMI have agonist activity at mammalian nAChRs at a concentration of 1 µM, which is lower than the concentration one would predict from their binding affinities.

The peak of Ca^2+^ influxes and the proportions of neurons that were excited did not depend on the dose of nicotine, ACE, or IMI. Rather, it exhibited an all or none response. Although the nAChR-dependent increase of intracellular Ca^2+^ may be mainly mediated by Ca^2+^ entry through nAChRs [27], the involvement of other calcium channels is also feasible. The nAChR-mediated Ca^2+^- influx activates voltage-dependent calcium channels (VDCCs) and Ca^2+^-uptake via VDCC augments the primary Ca^2+^ signals generated by nAChRs [27], [28], which may underlie the all or none response. However, the exact mechanism underlying this response needs further investigations to be fully understood.

### Differences between the effects neonicotinoids and nicotine on neuronal excitation

Firing evoked by nicotine or ACE (10–100 µM) rapidly rose and fell, whereas firing evoked by ACE (1 µM) or IMI rapidly rose but gradually fell, probably because of differential in desensitization potential to nAChRs. It is well known that nAChRs can undergo desensitization, which is a reversible reduction in response, even within a second of agonist applications at low concentrations of the agonist. Although the role of desensitization in the effects of nAChRs remains unclear, it has been proposed that desensitization can modulate the cholinergic activity of nAChRs [29], and chronic exposure to agonists can inhibit the normal actions of ACh at nAChRs via desensitization [15]. The peaks of the Ca^2+^ influxes induced by and the proportions of the neurons excited by ACE and IMI were somewhat lower than those by nicotine. Accordingly, there may be some differences among nicotine, ACE, and IMI in their agonist effects at nAChRs.

### Lack of a KCl-response after administrations of nicotine, ACE, or IMI

As shown in Figure 4A–C, applications of nicotine or the two neonicotinoids significantly decreased the effects of KCl (100 mM) on cerebellar neurons, even after they were removed by washing. As mentioned above, nAChR-mediated Ca^2+^- influx by nicotine, ACE, or IMI can activate VDCC. Because it has been reported that KCl-evoked Ca^2+^ permeability is coupled to VDCCs [28], [30], the initial uptake of Ca^2+^ ions via VDCC may serve as a negative feedback signal and elicit a transition of VDCC into a non-conducting inactivated state [31]. These ideas suggest that nAChR-mediated Ca^2+^-influx by nicotine, ACE, or IMI activates VDCC, which is followed by inactivation of VDCC and an attenuation of the KCl response. At 100 µM of nicotine, strong desensitization of nAChRs may activate some VDCC and subsequently induce relatively large responses by KCl. The precise mechanisms mediating these phenomena are unknown at present.

### Involvements of nAChR subtypes

As the three nAChR antagonists significantly inhibited the Ca^2+^ influxes in neurons induced by ACE and IMI, it is likely that ACE and IMI have direct agonist activity at nAChRs in cerebellar neurons. Complete blockade of the effects of all three drugs by the homomeric nAChR antagonist α-BT suggests that the response is mediated by the α7 receptor subtype, but the heteromeric nAChR antagonist DHβE also blocked the response, unexpectedly. These discrepant results are hard to interpret and may be attributable to unknown and combined responses between the heteromeric and homomeric nAChRs. MEC partially inhibited IMI-evoked responses, and DHβE partially inhibited nicotine-evoked responses. Therefore, ACE, IMI, and nicotine have somewhat different agonist activities at several types of nAChRs in cerebellar neurons.

In neonatal cerebellar granule cells, G-protein coupled muscarinic AChRs (mAChRs) have been detected [32]. ACh acts at both mAChRs and nAChRs, and Ca^2+^ ions are involved in the balance between mAChR and nAChR function [33]. Intracellular Ca^2+^ stores, which are modified by nAChRs, regulate mAChR stimulation. It has been reported that perinatal exposure to nicotine induces alterations in mAChRs [34], [35]. Further investigations are needed to fully elucidate the effects of neonicotinoids on mAChRs.

### The importance of nAChRs during development and adverse effects of nicotine

As described above, transient but significant expression of nAChRs during the perinatal stage is known to be important for brain development [14], [15]. In the developing brain, α4β2 and α7 subtypes of the nAChR have been implicated in neuronal proliferation, apoptosis, migration, differentiation, synapse formation, and neural-circuit formation [14], [15]. Accordingly, nicotine and neonicotinoids are likely to affect these important processes when it activates nAChRs. Accumulating evidence suggests that chronic exposure to nicotine causes many adverse effects on the normal development of a child [15], [36], [37]. Perinatal exposure to nicotine is a known risk factor for sudden infant death syndrome [38], low-birth-weight infants [39], and attention deficit/hyperactivity disorder [40]. Recent studies reported that gestational nicotine exposure modulates the cell-adhesion and cell-death/survival systems in the brains of adolescent rats and may lead to numerous behavioral and physiological deficits [41], [42].

It is known that newborn rats are equivalent to the human embryo from the aspect of brain development. The maturation of the human cerebellum takes about 36 weeks (from four to 39 weeks) *in utero*, whereas the maturation of the rat cerebellum takes only 30 days (from the 12-day embryo to P19) [43]. Thus, the effects of the neonicotinoids on neonatal rat cerebellar cultures imply that there may well be prenatal adverse effects of neonicotinoids in humans.

Studies of the *in vitro* absorption of IMI [44] and ACE [45] using the human intestinal cell line suggest that these neonicotinoids are also absorbed *in vivo* by active transporters in the intestines. An *in vivo* study revealed that ACE and IMI readily pass through the blood-brain barrier [46]. Furthermore, in mammals, some metabolites of the neonicotinoids show high affinities for mammalian nAChRs that are similar to those of nicotine [2]. These findings collectively suggest that these neonicotinoids may be potent risks to human health.

### Conclusions

The present study is the first to show that ACE, IMI, and nicotine exert similar effects at mammalian nAChRs. Based on our results, we suggest that excitation or desensitization or both of nAChRs by neonicotinoids may affect the developing mammalian nervous system, as is known to occur with nicotine. Further investigation is required to clarify the mechanisms of action of these substances and to determine safe concentrations for their application to agricultural crops as pesticides.

## Materials and Methods

### Animals and ethics statement

Sprague-Dawley rats (Clea Japan, Inc., Tokyo, Japan) were used in all experiments. All experiments were carried out in accordance with a protocol (ID: 23-10) approved by the Care and Use of Animals of Tokyo Metropolitan Institute of Medical Science, and all efforts were made to minimize the number of animals used and their suffering.

### Cerebellar cultures

The details of the culture methods have been described previously [47]. Briefly, the cerebella of P1 neonatal rats were digested with papain, and dissociated cells were suspended in a synthetic medium containing 1% fetal calf serum. The cells were plated at a density of 2.5×10^5^ cells/0.2 ml on a glass-bottomed dish (35-mm dish, 14-mm coverglass, Mat Tek Co., Ashland, MA, USA) that was pre-coated with 0.1 mg/ml poly-L-lysine (Sigma-Aldrich) and 10 µg/ml laminin (BD Biosciences, Franklin Lakes, NJ, USA). After 2 days, the medium was replaced with serum-free synthetic medium to prevent the growth of astrocytes. The serum-free synthetic medium consisted of Dulbecco's modified Eagle medium/F12 (Gibco®, Invitrogen, Carlsbad, CA, USA) with 10 µg/ml bovine insulin (Sigma-Aldrich), 100 µg/ml transferrin (Sigma-Aldrich), 30 nM sodium selenite, 5 nM thyroxine, 100 µM putrescine (Sigma-Aldrich), and penicillin-streptomycin (100 units/ml and 100 mg/ml, respectively, Gibco). One-half of the culture medium was replaced with a fresh medium every 3–4 days for the 16 DIV.

### Chemicals

Nicotine ((-)-nicotine) was purchased from Sigma-Aldrich (St Louis, MO, USA) at purities >99%. The neonicotinoid insecticides 1-(6-chloro-3-pyridylmethyl)-N-nitroimidazolidin-2-ylideneamine (IMI, purity >98%) and (E)-N^1^-[(6-chloro-3-pyridyl) methyl]-N^2^-cyano-N^1^ -methylacetamidine (ACE, purity >98%) were purchased from Kanto Chemicals Inc. (Tokyo, Japan) and Wako Chemicals Inc. (Osaka, Japan), respectively. Their chemical structures are shown in Figure 2A–C. They were dissolved in dimethyl sulfoxide (DMSO, Sigma-Aldrich), and their stock solutions (100 mM) were frozen at −30°C immediately prior to use to minimize their inactivation and degradation. The nAChR antagonists MEC (Sigma-Aldrich), α-BT (Calbiochem®, Merck, Darmstadt, Germany), and DHβE (Sigma-Aldrich) [48], [49] were used.

### Detection of nAChR subunit mRNA using RT-PCR

Expression of the α3, α4, and α7 nAChR subunits in cerebellar cultured cells was examined by RT-PCR. The primer sequences were essentially the same as those used by Moccia et al. [50]. The primer sequences, annealing temperature, and predicted product sizes are presented in Table 1. Expression of GAPDH was used as a positive control. Total RNA was extracted from the cerebellar cultures at 14 and 16 DIV and renal fibroblast cultures using the RNeasy Mini Kit (Qiagen, Tokyo, Japan). Using 2.5 µg of total RNA, RT-PCR was performed with RT-Ace (Toyobo, Tokyo, Japan) according to the manufacturer's protocol. PCRs for the α3 (L31621), α4 (L31620), and α7 (L31619) subunits and GAPDH (BC082592) were carried out using 0.5 µl of each reverse-transcribed solutions and AmpliTaq Gold (Applied Biosystems, CA, USA). In the PCR, the α3, α4, and α7 subunits were amplified over 40 cycles. GAPDH was amplified over 25 cycles.

**Table 1:** PCR primer sequences.

Subunit(Accession No.)	Primer Sequences (5′-3′)	Annealing Temperature(°C)	Product Size, bp
α3 (L31621)	FWD: GAC AAG ACC AAA GCT CTA CTC AAG TAC	65	435
	REV: GCA CAG AGA TGC AGA GTG TCA CCT TCT C		
α4 (L31620)	FWD: GCC ATC TAT AAG AGC TCC TGC AGC ATC	55	359
	REV: CTT CTC GCC AAA CTC TGA AGG CAG ATA G		
α7 (L31619)	FWD: GAC ATT CTC CTC TAT AAC AGT GCT GAT G	60	405
	REV: CTG AAA TGA GTA CAC AAG GGA TGA GCA G		
GAPDH (BC082592)	FWD: ACC ACA GTC CAT GCC ATC AC	55	451
	REV: GAT GTG GAT GAA GAA GTG TTT GC		

FWD, forward; REV, reverse.

Renal fibroblast cultures were used as a negative control. The culture methods were modified from Fu et al. [51]. Briefly, the cortices of adult rat (SD) kidneys were collected, minced, and cultured in culture flasks (NUNC, Roskilde, Denmark) using DMEM containing 10% FBS. After 2–3 weeks, propagated cells were dissociated with 0.25% trypsin, resuspended with culture medium, and cultured for a further 2–12 weeks. Before passage, the culture flasks were shaken to detach the more weakly adherent macrophages. More than 90% of the adherent cells were renal fibroblasts as identified by their elongated morphology and that they were positive for chicken anti–fibronectin (Abcam, Cambridge, MA) and mouse monoclonal anti-α-smooth muscle actin (Sigma-Aldrich) antibodies. The cells were used for the experiments after passage 3.

### Immunohistochemistry

The cultured cerebellar cells were fixed with 4% paraformaldehyde in 0.1 M phosphate buffer for 20 min at room temperature or cold methanol for 5 min. For the detection of specific antigens, the following primary antibodies were used: mouse monoclonal anti-Tuj1 (Covance, Princeton, NJ, USA) as a neuronal marker, rabbit anti-L1 (kindly provided from Dr. Asou) [52] and rat monoclonal anti-L1 (Millipore, Billerica, MA, USA) as granule cell markers, rabbit anti-calbindin D28K (Millipore) as a Purkinje cell marker, and rabbit anti-glial fibrillary acidic protein (GFAP, DAKO, Denmark) as an astrocyte marker. For double labeling, the cells were incubated with primary antibodies against TuJ1 and L1 or GFAP, then biotinylated anti-mouse or rabbit (rat) IgG (Vector, Burlingame, CA, USA), and finally with avidin-rhodamine (Vector) and corresponding FITC -conjugated secondary antibodies (from different host species, Chemicon and Molecular Probes, Invitrogen). All of the stained cells were examined by confocal laser microscopy (Zeiss, LSM510Meta, Oberkochen, Germany).

### Intracellular Ca^2+^ imaging

For Ca^2+^ imaging, we used cerebellar cultures that were 14–16 DIV. The cultures were loaded for 30 min at 37°C with 4 µM Fluo-4 acetoxymethyl ester (Molecular Probes, Invitrogen) in BSS that contained 0.14 M NaCl, 5.4 mM KCl, 1.8 mM CaCl_2_, 5.5 mM glucose, and 20 mM 4-(2-hydroxyethyl)piperazine-1-ethanesulfonic acid (HEPES, pH 7.4). Just before the experiment, the stock solutions of nicotine and neonicotinoids were dissolved and diluted by BSS to contain the same concentration of DMSO (≤0.001%). Time-lapse images of Fluo-4 fluorescence ([Ca^2+^]_i_) in cerebellar cells at 25°C were obtained using a ZEISS 510 Meta confocal laser scanning microscope system. Fluo-4 fluorescence was excited at 488 nm using an argon laser and the emitted fluorescence was collected at >515 nm. The cultures were continuously perfused at a rate of 1 ml/min with BSS by a peristaltic pump (Gilson, Minipuls 3, Middleton, WI, USA), and this flow rate changes the external solution surrounding the cells within 1 min. For nicotine and the neonicotinoids, pressure application was used at a rate of 0.5 ml/min by syringe pump (New Era Pump Systems, Inc. NE-300, Farmingdale, NY, USA) under continuous perfusion. To minimize desensitization of the nAChRs by drug leaking from the pipette tip (diameter 70–100 µm), the pipette used for the pressure application was placed at a target position approximately 100 µm from the neuron, and a minimal air bubble was inserted to isolate the agonist from the solution. Images at 1.5-second intervals were collected both before and after exposure to 1–100 µM of the neonicotinoids or nicotine for about 600 seconds. Around 500 seconds after the neonicotinoids or nicotine were applied, KCl (100 mM) was added to the culture to stimulate the neurons. Changes in the Fluo-4 fluorescence intensities in single cells, over a 10-µm diameter circular region of interest, were analyzed using the MetaMorph image analyzing system (Molecular Devices, Sunnyvale, CA, USA).

The peak relative Ca^2+^ influxes were calculated from the average of the highest fluo-4 intensities in each excited cell (*n* = 20–30), which were determined from the mean background intensity over the 60 seconds immediately before the drug application.

The proportion of the neurons that were excited was measured by counting the neurons that exhibited significant Ca^2+^ influx intensities (defined as greater than two times the baseline intensity) within 3 seconds after the reagent was administered, using MetaMorph. The number of neurons per mm^2^ was measured for each experiment.

The total numbers of small neurons were measured by counting small round cells after positive staining by Tuj1or L1-after fixation, and the count excluded a few Purkinje cells or astrocytes.

### Antagonist assay

For the antagonist assay, Fluo-4-Ca^2+^ imaging was used, as described above. First MEC, α-BT, or DHβE in BSS was added to the Fluo-4-labeled culture. After 5 min, Ca^2+^ imaging was started and ACE, IMI, or nicotine was administered by pressure application to minimize drug leakage, as described above, under constant perfusion of each antagonist solution. Then, Ca^2+^ imaging was stopped and the cultures were washed completely with BSS for about 5 min. Subsequently, Ca^2+^ imaging was restarted, and the drugs were applied by pressure application under constant perfusion of BSS. The targeted position was marked so that the subsequent drug applications without the antagonists were at the same location. Images were acquired for both processes and analyzed by MetaMorph, as described above for Ca^2+^ imaging.

### Statistical Analyses

The data were analyzed statistically using a Student's paired t-test or analysis of variance (ANOVA). Post hoc comparisons were carried out using the Bonferroni/Dunn test. To verify that the data were normally distributed, the Kolmogorov-Smirnov- normality test was applied. Values were considered statistically significant at probability (*P)<0.05*. The data are presented as the mean ± the standard error of the mean (S.E.M.). Each experiment was replicated with a minimum of three independent dishes, and the actual number of replicates for each experiment is listed in the corresponding figure legend.
